# The Impact of Nandrolone Decanoate in the Osseointegration of Dental Implants in a Rabbit Model: Histological and Micro-Radiographic Results

**DOI:** 10.3390/ma14092258

**Published:** 2021-04-27

**Authors:** Saturnino Marco Lupi, Alessandra Nicole Sassi, Alessandro Addis, Ruggero Rodriguez y Baena

**Affiliations:** 1 Department of Clinical Surgical, Diagnostic and Pediatric Sciences, University of Pavia, 27100 Pavia, Italy; alessandranico.sassi01@universitadipavia.it (A.N.S.); ruggero.rodriguez@unipv.it (R.R.y.B.); 2 CRABCC, Biotechnology Research Centre for Cardiothoracic Applications, 26027 Rivolta d’Adda, Italy; alessandro.addis@gmail.com

**Keywords:** dental implant, nandrolone decanoate, osseointegration, animals

## Abstract

Despite high rates of osseointegration in healthy patients, complex cases present an increased risk of osseointegration failure when treated with dental implants. Furthermore, if immediate loading of the implants is used, maximizing the response of the host organism would be desirable. Anabolic steroids, such as Nandrolone Decanoate (ND), are reported to have beneficial clinical effects on various bone issues such as osteoporosis and bone fractures. However, their beneficial effects in promoting osseointegration in dental implant placement have not been documented. The study aimed to examine histological changes induced by ND in experimental dental implants in rabbit models. Two dental implants were placed in the tibias of 24 adult rabbits. Rabbits were allocated to one of two groups: control group or test group. Rabbits in the latter group were given nandrolone decanoate (15 mg/kg, immediately after implant placement and after 1 week). Micro-radiographic and histological analyses were assessed to characterize the morphological changes promoted by the nandrolone decanoate use. Total bone volume and fluorescence were significantly higher in the control group after 2 weeks. Such a difference between the two groups might indicate that, initially, nandrolone lengthens the non-specific healing period characteristic of all bone surgeries. However, after the beginning of the reparative processes, the quantity of newly formed bone appears to be significantly higher, indicating a positive stimulation of the androgen molecule on bone metabolism. Based on micro-radiology and fluorescence microscopy, nandrolone decanoate influenced bone regeneration in the implant site. The anabolic steroid nandrolone decanoate affects the healing processes of the peri-implant bone and therefore has the potential to improve the outcomes of implant treatment in medically complex patients.

## 1. Introduction

Since Branemark’s discovery of osseointegration in the 1960s [1], scientific research has turned its interest towards aspects that could allow an enlargement of the initial indications: modifications of implants [2,3,4] and biofunctionalization of surfaces [5].

A different approach is explored in this study, namely the enhancement of the host’s response to implant placement. Indeed, despite the very high initial success rate in osseointegration [6], implant therapy has limitations in medically complex patients such as those suffering from systemic pathologies that have an effect on bone metabolism and on the potential for osseointegration, as in the case of autoimmune disease, diabetes mellitus, drug therapy, and past radiation therapy [7].

In the case of immediate, early, and late loading of an implant, a decisive factor for success is linked to the ability and the rate of bone tissue healing.

Many systemic drugs have been studied to enhance the osseointegration process of implants: parathyroid hormone, calcitriol, prostaglandin EP4 receptor agonist, dickkopf-1 antibody, sclerostin antibody, estrogen replacement and calcitonin, alendronate, bisphosphonate ibandronate, zoledronic acid, osteoprotegerin, and simvastatin [8,9,10,11,12,13,14,15,16,17,18,19,20,21,22,23,24,25,26,27,28,29].

To accelerate bone healing, the administration of Nandrolone Decanoate (ND) has been proposed in orthopedic settings for the initial treatment of patients with multiple fractures [30]. Calcium metabolism and ND have been demonstrated to have a synergic effect on increasing bone mineral density; they increase bone formation and stimulate the production of extra-osseous collagen while decreasing bone resorption [31].

ND is a doping molecule, banned for over 40 years in athletes. This improper and illicit use has led to the creation of an improper reputation for the molecule [32].

However, nandrolone has been used for the medical treatment of chronic diseases associated with a catabolic state such as burns, cornea healing, and osteoporosis [33,34], treatment of breast and prostate cancers [35,36], and, for its anti-anemic effects, treatment of male dialysis patients over the age of 50 [37]. ND is used in clinical practice to increase bone mineral mass, reduce bone loss, and decrease the incidence of fractures, with particular application to osteoporotic patients [38,39]. ND has been shown to promote in vivo fracture healing in mouse and rabbit models [40,41]. Anabolic steroids can stimulate osteoblastic activity, in which they eventually cause an increase in cortical bone density [42]. Nandrolone decanoate showed a favorable effect on osteogenic phenomena and probably an inhibitory action on bone resorption [43]. Not only do primary human osteoblasts possess androgen receptors, but also osteocytes, mononuclear, and endothelial cells of the bone marrow. In vitro studies were performed and have demonstrated how the proliferation and differentiation of osteoblastic cells can be stimulated by androgens. By inhibiting osteoclastic differentiation, androgens can also influence bone resorption [44,45]. The increasing bone mineralization linked to the decreasing bone resorption would seem independent of muscle function [46]. Administration of nandrolone induces an increased growth in the mandible of mouse models [47]. The drug also exhibits side effects. One of the major risks associated with taking nandrolone for long periods is hepatotoxicity and the related risk of liver cancer [48,49,50,51,52]. The carcinogenic risk of nandrolone could be linked to a sustained activity of stem cells which has been observed in different tissues [53].

The purpose of this study is to evaluate the effects of nandrolone decanoate administration on osseointegration in in vivo rabbit models. The null hypothesis of the study was that administration of nandrolone decanoate would not alter peri-implant total bone volume and fluorescence at 2, 3, and 4 weeks in rabbits.

## 2. Materials and Methods

Twenty-four adult male New Zealand rabbits, aged 8 months, were used for the study. The animals were housed in individual cages with food and water ad libitum in standard conditions of light, temperature, and humidity at the Centralized Animal Housing of the Faculty of Veterinary Medicine at the University of Milan. Before the experimental procedure, animals had an acclimation and an observation period of 7 days.

All the experimental procedures were conducted following the Italian law D. Lg 116/92 for the use of animals in scientific research. The study received ethical approval from the Italian Ministry of Health.

### 2.1. Surgical Procedure

Before surgical procedures, a fasting period of 10 h was observed, and all animals received the same anesthetic and analgesic protocol. Preanesthesia consisted of an intramuscular injection of ketamine (25 mg/kg) and xylazine (2 mg/kg). Anesthesia was obtained via an oronasal mask with a mixture of sevoflurane 8% and saturated oxygen and maintained with 4.5% sevoflurane and saturated oxygen. Post-operative analgesia was provided for 5 days with flunixin (1 mg/kg every 12 h).

**Figure 1:**
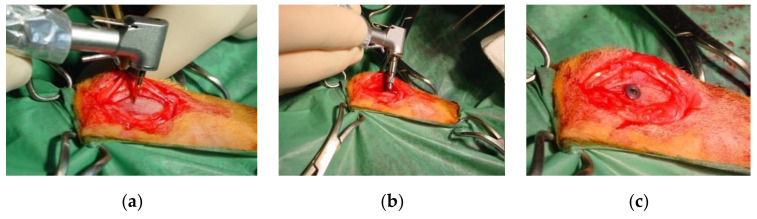
(**a**) Initial preparation of the implant site with lanceolate bur; (**b**) Implant placement; (**c**) Placement of the cover screw.

Surgery was performed under standard aseptic conditions following a standard procedure: the proximal tibial crest of the anesthetized animals underwent trichotomy, disinfection, and sectioning of the skin, subcutis, underlying muscles, and periosteum to expose the implant site. Each implant site was prepared with a standardized technique in the tibial proximal epiphysis; a lance-shaped drill mounted on a contra-angle was used (Figure 1) followed by drills of increasing diameter, until the size of the fixture was reached.

During all phases of bone site preparation, drills were cooled by continuous irrigation with a physiological solution in order to avoid bone overheating and consequent tissue necrosis.

A total of 48 sandblasted and acid-etched implants (LikeOs, Prodent Italia, Pero, Italy) with a diameter of 3.3 mm were inserted. After implant placement, the cover screw was secured to prevent the proliferation of bone or fibrous tissue.

**Figure 2:**
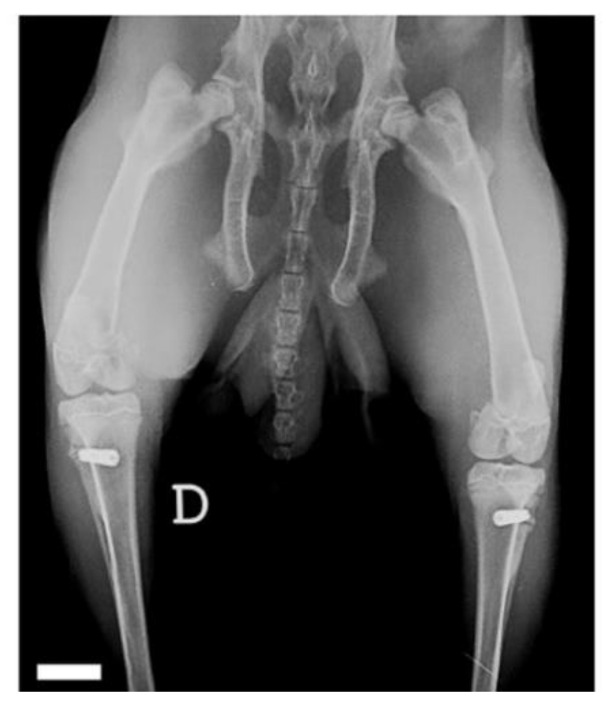
Final X-ray after implant placement surgery. Scale bar is 10 mm.

The periosteal and muscular planes were sutured with resorbable material (Poly-glactin 910, 4-0). The skin was sutured with non-absorbable monofilament (Nylon 4-0) and single stitches. In each rabbit, two implants were placed with the same procedure, one on each side. At the end of the operation, the rabbits, still under general anesthesia, underwent radiographic examination to assess the correct positioning of the fixtures and to be able to compare any migration or peri-implant bone resorption at the time of removal (Figure 2).

### 2.2. Experimental Procedure

The test group, consisting of four animals, was subjected to an injection of nandrolone immediately after implant placement and 7 days later. In this study, a dose of nandrolone decanoate of 15 mg/kg was administered. The control group, consisting of four animals, was subjected to a saline injection. Rabbits were sacrificed 2, 3 and 4 weeks after implant placement. Seven days before the suppression, the newly formed bone was marked with a subcutaneous injection of tetracycline (Oxytetracycline Hydrochloride, Sigma-Aldrich, St. Louis, MO, USA). This was injected subcutaneously in the back region through a dose of 30 mg/kg, previously diluted to 30 mg/mL in sterile saline under a fume hood. A venous blood sample was taken to evaluate the blood parameters on the day of the sacrifice. Biochemical investigations were carried out on the plasma and urine to evaluate the functionality of the various organs, apparatuses, and potential targets of action of nandrolone. The organs of the animals were subjected to histological examination to evaluate the presence of damage resulting from the administration of steroids.

### 2.3. Histomorphometric Analysis

The tibias were explanted, and then radiographic examination was carried out, which showed no signs of any migration or bone resorption phenomena.

All explanted pieces were fixed for 24 h in 10% formalin buffered at pH7 and then transferred to 50% alcohol. The sections were mounted onto double-sided tape on a plastic support and then cleaned and micro-radiographed. A section, obtained from the one mounted on the tape, was thinned up to 100 microns, polished, and photographed under a transmitted fluorescent light microscope.

The micro-radiographic images were analyzed by a Zeiss VIDAS using a 1 × 2 mm sample located at the border of the cortex on the endosteum side, at the neck of the im-plant, at the border with the oblique cortex for acute angle (excluding it from the measurement), and at the apex of the implant. Trabecular Bone Volume (TBV) was measured and expressed as the percentage of bone in the space of 1 × 2 mm or less. The fluorescence of bone tissue adjacent to the implant was measured in a sample of 1 mm of thickness and 2 mm^2^ of extension (the 1 × 2 mm grid) and placed at the periosteal margin of the cortex both at the collar and at the apex. The measurement was carried out on images of 1280 × 960 pixels corresponding to images of 2667 × 2000 microns for which the grid corresponded to 480 × 960 pixels = 460.6 kPixels. The amount of fluorescence was expressed in kPixels.

## 3. Results

**Figure 3:**
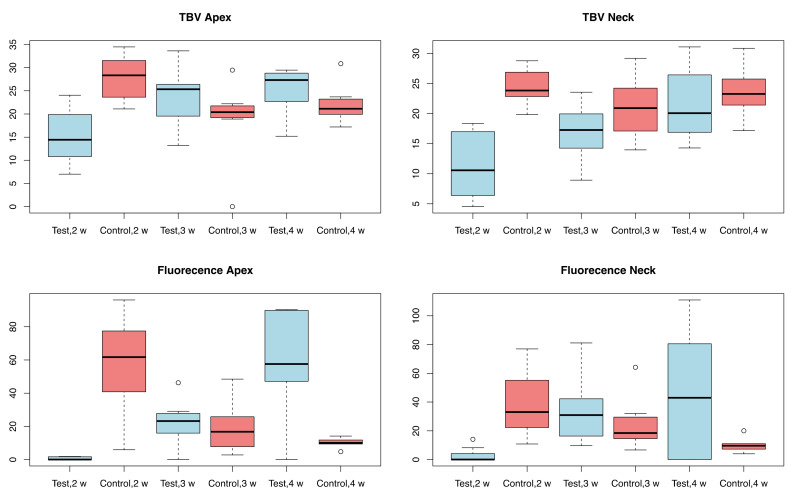
Boxplot of the results of the micro-radiographic and fluorescence analysis; w = weeks.

**Figure 4:**
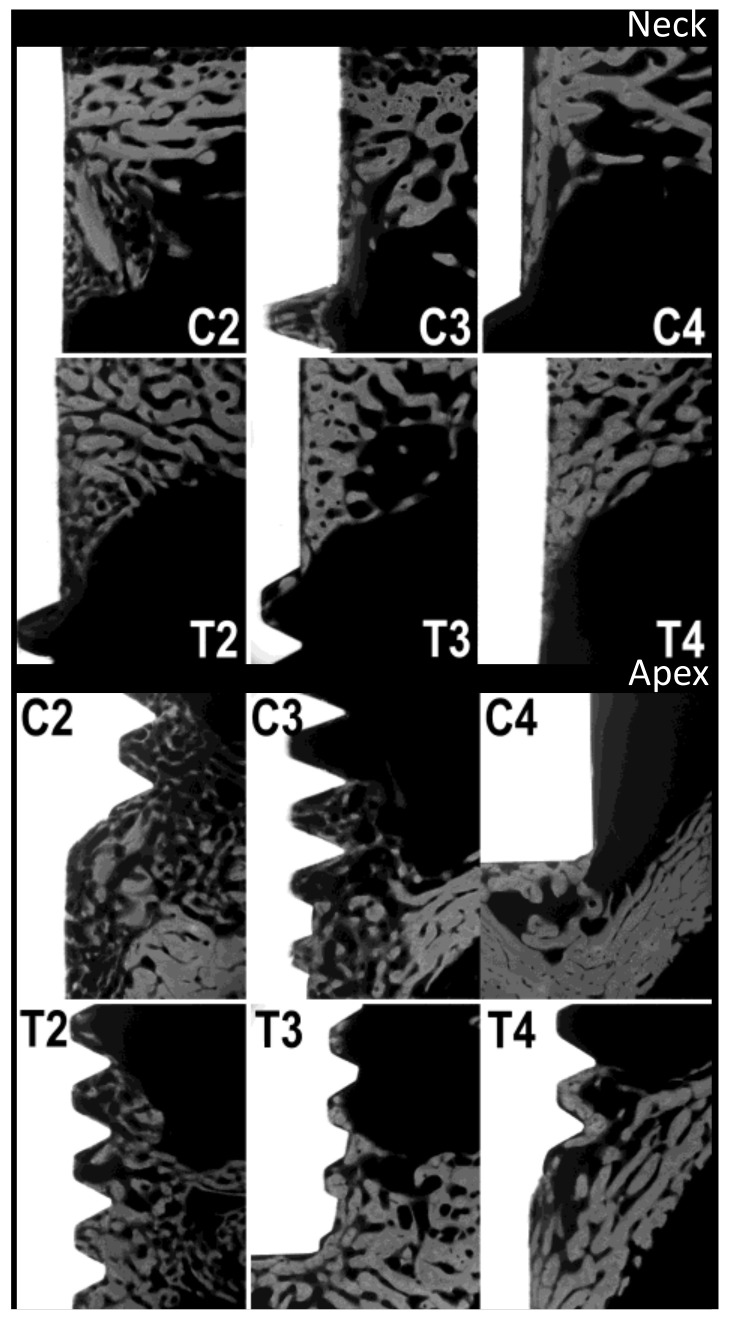
Micro-radiographies. C2, C3 and C4: control group at 2, 3 and 4 weeks, respectively. T2, T3 and T4: test group at 2, 3 and 4 weeks, respectively.

**Figure 5:**
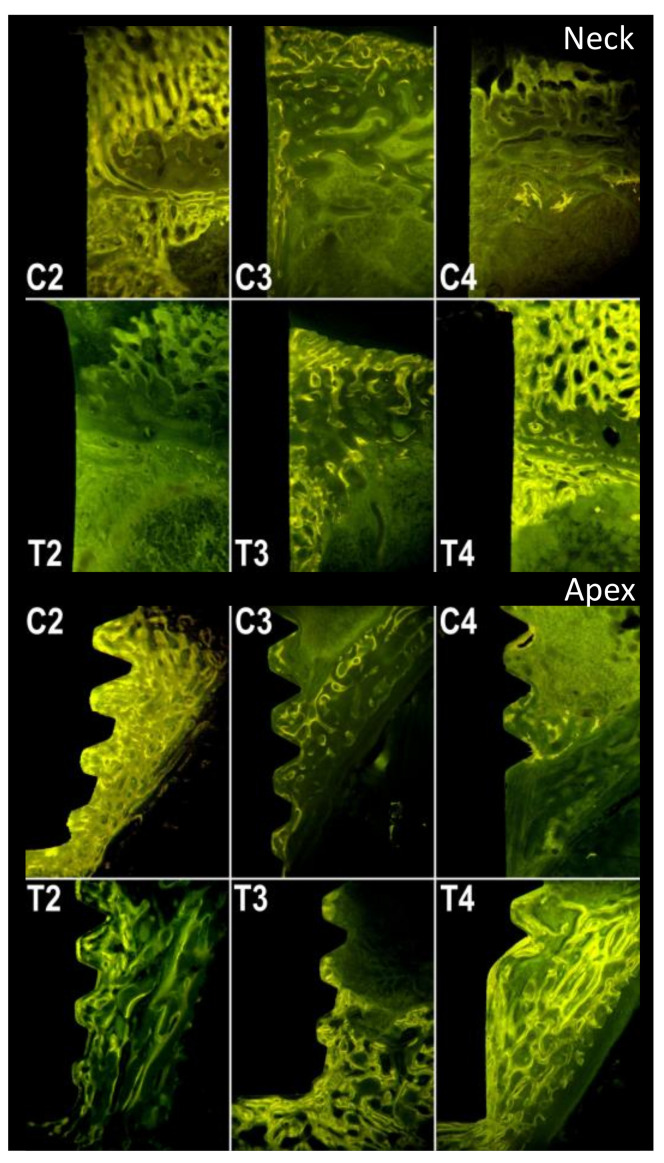
Fluorescence. C2, C3 and C4: control group at 2, 3 and 4 weeks, respectively. T2, T3 and T4: test group at 2, 3 and 4 weeks, respectively.

Four rabbits from the control group and four rabbits from the test group were sacrificed at each stage, i.e., at 2, 3 and 4 weeks. The rabbits used in the study weighed 3495 ± 145 g. The organs of the test group showed no steroid-related injuries. The results of the analysis with micro-radiography and fluorescence are shown in Table 1 and in the boxplot in Figure 3. Figure 4 and Figure 5, respectively, show representative images of the radiographic and fluorescence appearance of the examined groups.

Demineralized samples of the tibia from rabbits in the test group, microscopically analyzed in natural light and fluorescence, showed an increased amount of newly formed bone tissue.

Measurements of the micro-radiographs (Figure 3 and Figure 4) showed that the control group had the greatest bone production after 2 weeks and demonstrated a slight decrease afterwards, with no particular differences between collar and apex. In contrast, the test group had less bone production after 2 weeks, but this gradually increased after 3 and 4 weeks, with little difference between neck and apex.

The fluorescence (Figure 3 and Figure 5) highlights the different growth patterns. In the control group, this was high after 2 weeks, then drastically decreased after 3 weeks, and even more after 4 weeks. In the treated group, there was almost no fluorescence after 2 weeks, then it increased significantly after 3 weeks and even more after 4 weeks.

## 4. Discussion

The dose used in this study was chosen using previous research as a reference [54,55]. Indeed, no side effects were observed in these studies and this was further verified in the present work.

The results of our study indicate a positive androgenic osteogenic effect. They also show an initial negative effect on bone deposition. Bone formation certainly occurred after the administration of tetracycline in the test group sacrificed after 2 weeks, indeed, between 10 and 14 days. On the other hand, the control group probably started earlier, around 5 to 10 days. The bone was marked when the fluorochrome was administered on the 7th day. The high increase in fluorescence, and therefore in the newly formed bone, 1 week before sacrifice, allows us to assume a possible further amplifying effect on the amount of bone that would be deposited after 4 weeks, while the controls would likely show only a reduction in skeletal mass. The results, therefore, indicate that androgens could have an enhancing effect on osteogenic activity, but also on macrophage and reconstructive activity. This involves many non-specific phenomena that follow surgery, prolonging healing over time.

The results, therefore, indicate that androgens should not be used immediately, but, in rabbits, at least 1 week after surgery and eventually after 15 and 30 days. The enhancement of post-operative non-specific phenomena by androgens could explain why results are sometimes not different from those of the controls, and sometimes even negative. These effects would be even more pronounced if androgens used were more powerful. Given the obtained results, it is suggested that further experiments providing ND, should be conducted at a later stage. The histomorphological study of the sections of the bone segments containing the implants, included without decalcification in methyl methacrylate, showed, in the control animals, the presence of good amounts of newly formed trabecular bone 2 weeks after the insertion of the implants. The newly formed bone was abundant near the cortices, and at the apex and collar of the implants, but was also found along the body of the implants themselves. Fluorescence was conspicuous throughout the newly formed bone in control animals sacrificed 2 weeks after implant placement. On the other hand, when animals were sacrificed at 3 and 4 weeks after implant placement, the amount of newly placed bone was drastically reduced and limited to the apexes and necks. Fluorescence was poorly detectable. In animals treated with nandrolone, the amount of newly formed bone was significantly lower than the one found in control animals 2 weeks after the insertion of the implants. This bone, still visible in good quantity, was, however, found almost exclusively at the apexes and necks of the implants. It was also poorly fluorescent. Contrary to the control group, animals treated with nandrolone showed a gradual increase in the newly formed bone surrounding the implants both 3 and 4 weeks after their insertion. As far as the fluorescence is concerned, the newly formed bone, after 3 weeks, showed a conspicuous level of fluorescence, which further increased after 4 weeks to almost match the level found in the bone of the control animals sacrificed 2 weeks earlier.

The rabbit model has been previously used by researchers to study the effect of systemic drugs on osseointegration processes. Almagro et al. reported similar results to the present study using a human parathyroid hormone; however, in their case, the drug was administered after 12 weeks, indicating a longer healing time [11].

The use of bisphosphonates was also analyzed, producing non-unique results. Therefore, further studies are required on the subject. For example, Chacon et al. examined the use of alendronate without observing any histologic effect on osseointegration after 6 weeks [23]. Li et al. found that systemic treatment with zoledronic acid could efficiently promote the early bone healing of implants in the autogenous grafted bone of osteoporotic rabbits by increasing the early osseointegration and fixation of implants at 2 and 8 weeks [15]. Conversely, Kim et al. found that administration of bisphosphonates interfered with normal bone remodeling after tooth extraction and long-term healing around the implants was impaired, concluding that patients taking bisphosphonates should be treated with caution when performing tooth extraction or placing dental implants [56].

The results of the present study exhibit large values of standard deviation, particularly in fluorescence. This indicates large possible variations around the mean. The deviation may be due to the small number of individuals taken into account. For this reason, the authors believe that a larger experiment could be beneficial. Furthermore, the results could have been influenced by the choice of the area in which fluorescence was measured. To the best knowledge of the authors, there are no studies that considered this issue, impeding further discussions.

The results of the present study have shown the real stimulation, by an anabolic hormone (nandrolone), on the bone healing process after surgery for the insertion of dental endosseous implants. The process of bone neo-apposition also appears to have increased later than in the control animals that had not received the administration of nandrolone. This indicates that nandrolone probably acts at first by lengthening the non-specific healing period characteristic of all bone surgeries. However, when the reparative processes begin, the quantity of newly formed bone appears to be decidedly higher, indicating a positive stimulation of the androgen molecule on bone metabolism.

The blood tests performed on the animals used in the experiment did not show any interference of nandrolone on the haemato-chemical values chosen to evaluate any metabolic abnormalities.

Histological examinations performed after autopsy examination of the euthanized rabbits did not reveal any macro or microscopic alteration of the anatomical tissues examined.

## 5. Conclusions

In conclusion, the results in this animal model suggest a possible stimulation of osteogenic processes by androgens, but also underline a side effect that could have been the cause of the alternating results obtained in previous research. The results would indicate that, in control animals, there was an active bone deposition around the 7th day from the insertion of the implants. They would, therefore, indicate that the non-specific phenomena of repair due to surgery and the insertion of the implants would end after 6 or fewer days. On the contrary, in treated animals, the non-specific repair period appears to be increased by androgens and the deposition of new bone would only appear later, 15 days after surgery. Androgens, however, have an amplifying effect later on, stimulating osteogenesis activities.

## Figures and Tables

**Table 1:** Micro-radiography and fluorescence results.

**TBV NECK**				**TBV APEX**		
WEEK	TEST	CONTROL	*p*	TEST	CONTROL	*p*
2	11.35 ± 5.50	24.47 ± 2.90	****	15.17 ± 6.04	27.82 ± 4.731	***
3	16.93 ± 4.53	20.96 ± 5.04		23.67 ± 6.20	18.82 ± 9.012	
4	21.53 ± 5.93	23.66 ± 4.41		25.29 ± 5.09	22.2 ± 4.387	
**FLUORECENCE NECK**				**FLUORECENCE APEX**		
WEEK	TEST	CONTROL	*p*	TEST	CONTROL	*p*
2	2.786 ± 5.395	38.6 ± 22.54	***	0.647 ± 0.895	57.74 ± 28.93	****
3	33.76 ± 22.96	25.36 ± 19.09		22.51 ± 13.18	20.84 ± 14.85	
4	44.76 ± 44.41	10.25 ± 5.386		57.06 ± 33.86	10.04 ± 3.11	**

TBV: Total Bone Volume; *p*: **: <0.001, ***: <0.0001, ****: <0.00001.
